# Exploring the potential of BH3 mimetic therapy in squamous cell carcinoma of the head and neck

**DOI:** 10.1038/s41419-019-2150-8

**Published:** 2019-12-04

**Authors:** Rachel J. Carter, Mateus Milani, Michael Butterworth, Ahoud Alotibi, Nicholas Harper, Govindaraju Yedida, Georgia Greaves, Aoula Al-Zebeeby, Andrea L. Jorgensen, Andrew G. Schache, Janet M. Risk, Richard J. Shaw, Terry M. Jones, Joseph J. Sacco, Adam Hurlstone, Gerald M. Cohen, Shankar Varadarajan

**Affiliations:** 1 Liverpool Head and Neck Centre, Liverpool, L69 3GE UK; 2 Department of Molecular and Clinical Cancer Medicine, Liverpool, L69 3GE UK; 3 Department of Molecular and Clinical Pharmacology, Liverpool, L69 3GE UK; 4 Department of Biostatistics, University of Liverpool, Liverpool, L69 3GE UK; 5 Faculty of Biology, Medicine and Health, Division of Infection, Immunity and Respiratory Medicine, University of Manchester, Manchester, M13 9PT UK

**Keywords:** Cancer, Cell biology

## Abstract

Squamous cell carcinoma of the head and neck (SCCHN) is the sixth most common cancer worldwide, with overall survival of less than 50%. Current therapeutic strategies involving a combination of surgery, radiation, and/or chemotherapy are associated with debilitating side effects, highlighting the need for more specific and efficacious therapies. Inhibitors of BCL-2 family proteins (BH3 mimetics) are under investigation or in clinical practice for several hematological malignancies and show promise in solid tumors. In order to explore the therapeutic potential of BH3 mimetics in the treatment of SCCHN, we assessed the expression levels of BCL-2, BCL-X_L_, and MCL-1 via Western blots and immunohistochemistry, in cell lines, primary cells derived from SCCHN patients and in tissue microarrays containing tumor tissue from a cohort of 191 SCCHN patients. All preclinical models exhibited moderate to high levels of BCL-X_L_ and MCL-1, with little or no BCL-2. Although expression levels of BCL-X_L_ and MCL-1 did not correlate with patient outcome, a combination of BH3 mimetics to target these proteins resulted in decreased clonogenic potential and enhanced apoptosis in all preclinical models, including tumor tissue resected from patients, as well as a reduction of tumor volume in a zebrafish xenograft model of SCCHN. Our results show that SCCHN is dependent on both BCL-X_L_ and MCL-1 for apoptosis evasion and combination therapy targeting both proteins may offer significant therapeutic benefits in this disease.

## Introduction

Head and neck cancer, of which squamous cell carcinomas account for over 90% of diagnoses, is the sixth most common cancer worldwide^1^. Although etiological factors, such as tobacco use, alcohol consumption, and human papillomavirus (HPV) infection, as well as the mutation of key genes associated with SCCHN have been identified, this has not translated into better therapies and the prognosis for many HPV negative (HPV^−^) patients remains significantly poorer than for HPV positive patients^2–4^. With the currently preferred treatment modalities of surgery, radiotherapy and/or chemotherapy, local recurrence or metastasis is observed in >50% of HPV^−^ patients and is often associated with significant morbidity and mortality^5^, thus emphasizing the urgent need for improved therapeutic strategies. Until recently, the only targeted therapy approved for SCCHN was the monoclonal antibody, cetuximab, which inhibits the epidermal growth factor receptor (EGFR). Although EGFR is frequently overexpressed in SCCHN^4^, responses to cetuximab have been varied, and generally disappointing^4,6^. However, there is new promise for immunotherapy, with the recent FDA approval of the PD-1 specific antibody Pembrolizumab for first-line treatment of SCCHN^7^.

Evasion of apoptosis is one of the cardinal features of cancer. Most anticancer therapies facilitate cancer cell death by inducing the intrinsic pathway of apoptosis, which is regulated by the balance of antiapoptotic and proapoptotic BCL-2 family proteins^8^. High expression levels of antiapoptotic BCL-2 family members (BCL-2, BCL-X_L_, and MCL-1) allow apoptotic evasion in multiple malignancies, prompting the development of a novel class of inhibitors (BH3 mimetics) for these proteins^9^. The first BH3 mimetics, ABT-737 and ABT-263 (Navitoclax), inhibit BCL-2, BCL-X_L_, and BCL-w^10,11^, and were followed by more selective inhibitors, such as the BCL-2-specific Venetoclax (ABT-199), recently approved for treatment of chemorefractory chronic lymphocytic leukemia^12,13^, in addition to newly diagnosed acute myeloid leukemia, in combination with hypermethylating agents, such as azacytidine and decytabine^14^. Similarly, BH3 mimetics that specifically target either BCL-X_L_ or MCL-1 show promise in several hematological malignancies^15–19^. Studies utilizing BH3 mimetics in SCCHN have largely been limited to ABT-737, which reportedly synergizes with cisplatin, etoposide and radiation^20–22^.

Our aim was to assess the potential of BH3 mimetic therapy in SCCHN utilizing several preclinical models including explant cultures of surgically resected human tumors and a zebrafish xenograft model.

## Materials and methods

### Cell lines, primary cells, and explant culture

UM-SCC-1 (derived from an oral cavity primary tumor), UM-SCC-11B, UM-SCC-17A, and UM-SCC-17AS (all derived from larynx), UM-SCC-74A and UM-SCC-81B (derived from oropharynx) were obtained from T. Carey (University of Michigan), and authenticated by short tandem repeat (STR) profiling. Cell lines were cultured in DMEM with high glucose and GlutaMAX (Gibco, 32430-027), supplemented with 10% fetal bovine serum (FBS) (Life Technologies, 10270106) and 1× NEAA (Gibco, 11140-035), and grown in a humidified incubator at 37 °C and 5% CO_2_. Primary (low passage, <20) SCCHN cells LIV7K (derived from a T3N2bMx oral tongue primary tumor), LIV37K (T3N2bMx floor of mouth), and LIV72TS (T3N2c oral tongue) were generated following informed consent under REC EC47.01 (LIV7K) or 10/H1002/53 (LIV37K and LIV72TS) and cultured in KSFM (Gibco, 17005-034) supplemented with bovine pituitary extract and human recombinant EGF (Gibco, 37000-015). SCCHN primary tumor tissue (6 oral cavity, 1 larynx, and 3 oropharynx) was obtained from individuals undergoing surgery as a primary treatment, with informed consent under REC 10/H1002/53, cut into pieces of 1–2 mm^3^ and cultured in DMEM with high glucose and GlutaMAX, supplemented with 10% FBS, 100 U/ml penicillin, 0.1 mg/ml streptomycin (Sigma, P0781), and 2.5 µg/ml Amphotericin B (Gibco, 15290-026).

### Cloning and generation of H2B-mRFP-positive cells for zebrafish xenografts

Histone H2B-mRFP fusion protein cDNA was amplified from pHIV-H2B-mRFP (AddGene #18982) by polymerase chain reaction and transferred to a modified form of the lentiviral plasmid pLJM1 (AddGene #19319) and sequence-verified. To make H2B-mRFP-expressing lentivirus, 4 × 10^6^ HEK293T cells were transfected with the lentiviral packaging plasmids; pMD2.G (AddGene #12259) and psPAX2 (AddGene #12260) together with pLJM1 H2B-mRFP using polyethylenimine (1 mg/ml), and lentivirus harvested 72 h later. This was used for transducing UM-SCC-81B cells, in the presence of Polybrene (4 µg/ml) by spinoculation (2000 rpm, 1 h). Upon validation of nuclear expression of H2B, cells stably expressing H2B were selected by exposure to puromycin (1 µg/ml) for 2 weeks.

### Reagents

ABT-199 (Selleck, S8048), A-1331852 (a gift from Abbvie), S63845 (Apex Biotech Corporation, AB-A8737) and cisplatin (Abcam, ab141398) were used. Antibodies against BCL-2 (Cell Signaling Technology (CST) 4223), BCL-X_L_ (Santa Cruz, sc-8392), MCL-1 (Santa Cruz, sc-819), GAPDH (Santa Cruz, sc-25778) were used for Western blotting. For IHC, antibodies against BCL-2 (1:200, CST 15071), BCL-X_L_ (1:200, CST 2764), MCL-1 (1:50, Abcam, ab114026; 1:50, Proteintech, 16225-1-AP; 1:50, Sigma, HPA008455; 1:25, CST 39224) and cleaved PARP (1:50, CST 5625) were used. For transient transfections, siRNAs specific to BCL-2 (QIAGEN, SI00299411), BCL-X_L_ (QIAGEN, SI03025141), MCL-1 (QIAGEN, SI02781205), and a non-targeting control, siC (QIAGEN, 1027310) were incubated with Interferin siRNA transfection reagent (Polyplus transfection Inc.) and added to cells at a concentration of 10 nM for 72 h.

### BH3 profiling, apoptosis, and colony formation assays

BH3 profiling and apoptosis (assessed by measuring the extent of phosphatidylserine (PS) externalization) were performed as described previously^16^. For colony formation assays, a defined number of cells were exposed to different drugs and/or X-ray irradiation using a CellRad X-ray irradiator (Faxitron Bioptics) and incubated for 7–14 days. Cell colonies were fixed using 1:7 vol/vol acetic acid/methanol for 5 min and stained with 0.5% crystal violet (in 20% methanol) for 2 h, then counted on a GelCount colony analyzer (Oxford Optronix). Plating efficiency (PE) for the untreated controls and surviving fraction (SF) were determined using the formulae:

$$\begin{array}{l}{\rm{PE}} = \left[ {{{{\rm no.}\,{\rm of}\,{\rm colonies}\,{\rm formed}}}{\mathrm{/}}{{{\rm no.}\,{\rm of}\,{\rm cells}\,{\rm seeded}}}} \right]\,{\mathrm{and}}\\ {\rm{SF}} = \left[ {{{{\rm no.}\,{\rm of}\,{\rm colonies}\,{\rm formed}}}{\mathrm{/}}\left( {{{{\rm no.}\,{\rm cells}\,{\rm seeded} \times {\rm PE}}}} \right)} \right] \times 100.\end{array}$$

### Western blotting

Whole cell extracts were prepared in RIPA buffer containing cOmplete Mini protease inhibitor cocktail (Sigma, 11836170001) and MG-132 (20 µM) followed by centrifugation at 14,000 rpm at 4 °C for 10 min. Proteins were resolved on 10 or 15% sodium dodecyl sulfate polyacrylamide gel electrophoresis, transferred to nitrocellulose membranes (VWR, 10600002.P), incubated with the indicated primary and secondary antibodies and visualized using ECL reagents (GE Healthcare) and ChemiDoc (BioRad).

### Immunohistochemistry (IHC), image acquisition, and analysis

Explants, surgically resected from patients, were acclimatized overnight to the culture conditions before exposure to BH3 mimetics for 48 h. Explants were fixed in 4% paraformaldehyde at 4 °C for 24 h and infiltrated with paraffin using a HistoCore PEARL Tissue Processor (Leica). Following embedding in paraffin blocks, tissues were cut into 5 μm sections. Sections from explants and TMAs (generated in-house—clinical and pathological patient details in Table 1) were processed and stained using the Bond RX^m^ autostainer (Leica). Briefly, TMA sections were deparaffinized, subjected to heat induced epitope retrieval, endogenous peroxidase activity blocked, and then incubated with the relevant primary antibodies and a non-species-specific linker and HRP, which were detected using DAB + chromogen. Sections were counterstained with hematoxylin, dehydrated, cleared and mounted using EcoMount (Biocare Medical). Images were acquired at 20× magnification using a Nikon Eclipse E800 microscope for explants, or by scanning at 40× magnification using an Aperio slide scanner (Leica) for TMAs. Explant staining was quantified by counting the SCCHN nuclei in each image and determining the percentage of positively stained cells. TMA antibody staining intensity was analyzed using QuPath software^23^. The cytoplasmic DAB optical density was measured for each tumor cell within a tissue sample, and a histo-score (*H*-score; a quantitative measurement of the intensity of staining for a particular antibody) generated for that sample. The *H*-score was averaged between two or three tissue samples available per patient. Kaplan–Meier survival curves were generated using GraphPad Prism version 8.0.1 (GraphPad Software) and overall survival data from patients in the lower- and upper-most quartiles for expression (*H*-score) of each antiapoptotic protein.

**Table 1:** SCCHN patient cohorts.

	Oral Cavity *n* (%)	Hypopharynx *n* (%)	Larynx *n* (%)
*Age at diagnosis*			
<60 years	44 (45)	8 (40)	23 (41)
≥60 years	53 (55)	12 (60)	33 (59)
Unknown	0	0	18
*Gender*			
Male	68 (70)	14 (70)	51 (91)
Female	29 (30)	6 (30)	5 (9)
Unknown	0	0	18
*T stage*			
T1	7 (7)	4 (20)	1 (2)
T2	51 (53)	6 (30)	13 (23)
T3	10 (10)	6 (30)	22 (39)
T4	28 (29)	4 (20)	20 (36)
Unknown	1	0	18
*N stage*			
N0	35 (37)	4 (20)	29 (52)
N1	17 (18)	2 (10)	8 (14)
N2	42 (44)	14 (70)	19 (34)
N3	1 (1)	0 (0)	0 (0)
Unknown	2	0	18
*ECS*			
Yes	42 (44)	7 (37)	16 (29)
No^a^	54 (56)	12 (63)	39 (71)
Unknown	1	1	19
*Recurrence*^b^			
Yes	32 (42)	3 (15)	6 (12)
No	45 (58)	17 (85)	45 (88)
Unknown	20	0	23
*Survival at 5 years*			
Alive	36 (40)	10 (50)	27 (48)
Deceased	53 (60)	10 (50)	29 (52)
Unknown	8	0	18

Clinical and pathological characteristics of patients comprising oral cavity, hypopharynx and larynx tissue microarrays. *ECS* extracapsular spread.

^a^Includes those patients who are N0 (no nodal involvement).

^b^Recurrence data is to 10 years post-diagnosis for oral cavity, and 5 years for hypopharynx and larynx.

### Zebrafish xenografts

Zebrafish studies were completed under the approval of the University of Liverpool Animal Welfare and Ethical Review Body. Nacre *ubiq*:secAnnexinV-mVenus embryos^24^, obtained from The University of Manchester Biological Services Facility, were incubated at 28 °C in egg water (60 μg/ml Tropic Marin salt in distilled water) until 48 h postfertilization (hpf). Following cell implantation, fish were maintained in a humidified light-cycling incubator (14 h on, 10 h off) at 34 °C. For toxicity studies, embryos at 72 hpf were exposed to increasing concentrations of BH3 mimetics (at 34 °C) and mortality assessed up to 120 hpf. For xenograft studies, UM-SCC-81B cells stably expressing H2B-mRFP were injected into the pericardial cavity at 48 hpf, as previously described^25^. At 72 hpf, fish were screened for the presence of red fluorescent cell masses, following anesthetization using MS-222 (160 µg/ml for maximum 15 min) and images acquired using a Leica MZ16F microscope. Successfully xenografted fish were randomly placed individually in wells of a 24-well plate and either DMSO or BH3 mimetics added to 1 mM Tris-buffered egg water. Fish were euthanized by 120 hpf using MS-222 (250 µg/ml). Images of tumor masses were acquired and 2D tumor areas calculated using ImageJ software.

### Statistical analysis

Statistical analyses were conducted using GraphPad Prism version 8.0.1 (GraphPad Software) as described in the Figure legends. In all instances, *P < 0.05, **P < 0.01, ***P < 0.001.

## Results

### SCCHN cell lines express varying levels of anti-apoptotic proteins and are primed to undergo apoptosis

**Fig. 1:**
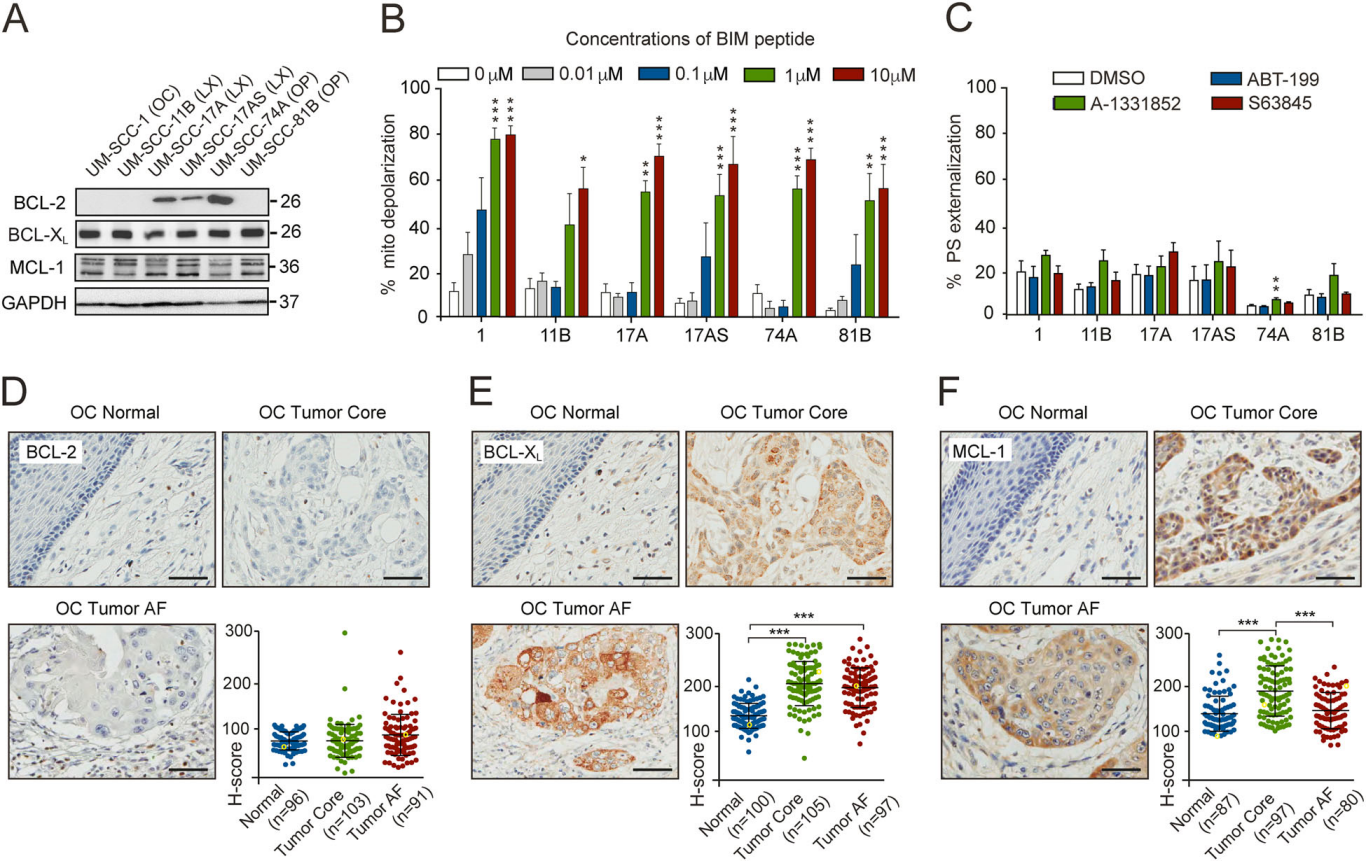
BCL-X_L_ and MCL-1 but not BCL-2 are highly expressed in SCCHN cell lines and oral cavity tissue microarrays from SCCHN patients. **a** Western blot analysis of the three major anti-apoptotic BCL-2 family proteins in SCCHN cell lines. Sites of primary tumors are indicated in brackets: OC oral cavity, LX larynx, OP oropharynx. **b** BH3 profiling with increasing concentrations of BIM peptide revealed that the indicated SCCHN cell lines were primed to undergo apoptosis. **c** SCCHN cell lines exposed to the indicated BH3 mimetics (100 nM) for 24 h failed to undergo apoptosis, as assessed by phosphatidylserine (PS) externalization. **d**–**f** Representative IHC images of oral cavity (OC) normal tissue, primary tumor core and advancing front (AF), stained using antibodies against **d** BCL-2 **e** BCL-X_L_, and **f** MCL-1 (clone D5V5L) and counterstained with hematoxylin. Scale bars 50 μm. Dot plots depict the relative staining intensities (*H*-scores) of the specified antibody. Each dot represents the data from an individual patient. The IHC images shown correspond to the yellow circles in the graph. **b**, **c** One-way ANOVAs with Dunnett’s multiple comparisons tests, with a single pooled variance. Error bars = mean ± SEM of at least three independent experiments. **d**–**f** Normality tests were performed, followed by Kruskal Wallis tests with Dunn’s multiple comparisons tests. Error bars = mean ± SD. *P < 0.05, **P < 0.01, ***P < 0.001.

Given the poorer clinical outcome associated with HPV^−^ SCCHN, we wished to explore the potential of BH3 mimetic therapy in this disease. We first determined the expression profile of the major antiapoptotic BCL-2 family proteins in six HPV^−^ cell lines, derived from different head and neck subsites. MCL-1 and BCL-X_L_ were expressed in all the cell lines, whereas BCL-2 was expressed in only 3 of the 6 cell lines tested (Fig. 1a). To ascertain the responsiveness of SCCHN cell lines to apoptotic stimuli, BH3 profiling was utilized by exposing cells to increasing concentrations of BIM peptide, which binds promiscuously to all anti-apoptotic BCL-2 family members^26^. All cell lines exhibited a concentration-dependent increase in mitochondrial membrane depolarization (Fig. 1b), suggesting they were all primed to undergo apoptosis.

### BH3 mimetics, as single agents, do not induce apoptosis in SCCHN cell lines, nor do they sensitize cells to cisplatin or irradiation

None of the specific BH3 mimetics, ABT-199 (BCL-2 specific^12^), A-1331852 (BCL-X_L_ specific^15^), or S63845 (MCL-1 specific^17^), induced pronounced apoptosis as single agents (Fig. 1c). Moreover, none of the BH3 mimetics synergized with cisplatin or irradiation in significantly reducing the clonogenic potential of the SCCHN cell lines tested, except UM-SCC-1 cells, which showed synergism between A-1331852 and either cisplatin or radiotherapy (Supplementary Figs. S1 and S2).

### IHC of SCCHN tumor tissue reveals high expression of BCL-X_L_ and MCL-1 but little/no BCL-2

Next, we examined the expression of BCL-2, BCL-X_L_, and MCL-1 in TMAs comprising tissue obtained from 191 SCCHN patients at the time of surgery, from oral cavity, hypopharyngeal and laryngeal tumors. The patient population was representative of the typical spectrum of HPV^−^ SCCHN (Table 1). The TMAs included tissues classified as core (center of the primary tumor), advancing front (invasive edge of the tumor) and normal (non-malignant adjacent tissue). IHC was performed using antibodies validated by Western blotting and IHC in SCCHN cell lines in which the anti-apoptotic proteins were downregulated with RNAi (Supplementary Fig. S3). A growing literature surrounds the use of antibodies that are not carefully validated for IHC analysis, which result in inconsistent, and often misleading, correlations with patient outcome^27,28^. While the antibodies against BCL-2 and BCL-X_L_ exhibited specificity in both Western blots (characterized by a specific band at the predicted molecular weight) and IHC (characterized by specific mitochondrial staining, which decreased following siRNA transfections), problems were evident with the different MCL-1 antibodies used (Supplementary Fig. S3). Some antibodies detected multiple isoforms of MCL-1, whereas others were specific to the predominant, antiapoptotic isoform^29^. IHC revealed differing staining patterns of MCL-1, ranging from nuclear localization to cytosolic/mitochondrial staining (Supplementary Fig. S3). Using RNA interference and specific mitochondrial staining as primary indicators of specificity, we deemed clone D5V5L the most specific MCL-1 antibody, which was used in all subsequent experiments. TMAs were stained with the appropriate antibodies and protein expression levels scored according to the staining intensity, denoted by the histo-score (*H*-score) (Fig. 1d–f).

**Fig. 2:**
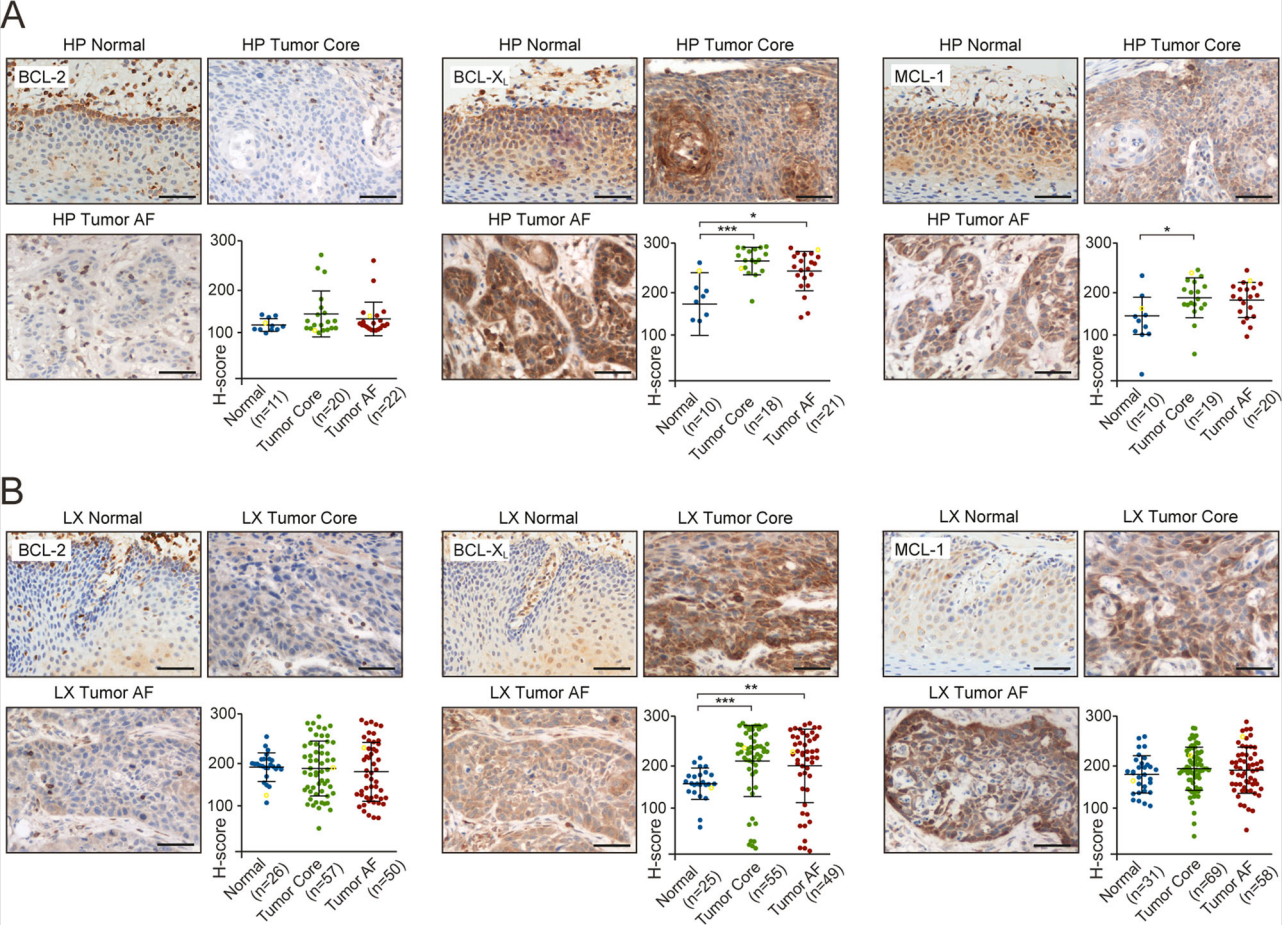
BCL-X_L_ and MCL-1 but not BCL-2 are highly expressed in hypopharynx and larynx tissue microarrays from SCCHN patients. Representative IHC images of normal tissue, primary tumor core and advancing front (AF) from **a** hypopharynx (HP) and **b** larynx (LX) stained against the indicated antibodies and counterstained with hematoxylin. Scale bars 50 μm. The plots depict the relative staining intensities (*H*-score) of the specified antibody. Each dot represents the data from an individual patient. The IHC images shown correspond to the yellow dots in the graph. Normality tests were performed, followed by Kruskal Wallis tests with Dunn’s multiple comparisons tests. Error bars = mean ± SD. *P < 0.05, **P < 0.01, ***P < 0.001.

Expression of BCL-2 was low in all normal and tumor tissue from the oral cavity and hypopharynx (Figs. 1d and 2a), whereas it appeared to be more highly expressed in the tumor core, advancing front and in the adjacent normal tissues of the larynx (Fig. 2b). BCL-X_L_ expression was significantly elevated in the tumor core and advancing front of oral, hypopharyngeal and laryngeal SCCHN, compared to adjacent normal tissues (Figs. 1e and 2a, b). MCL-1 was highly expressed in the tumor core from the oral cavity compared to the normal tissue, whereas this was not evident in the advancing front (Fig. 1f). MCL-1 expression was generally higher in the tumor core and advancing front of hypopharynx, compared to the normal tissues, whereas in the larynx, MCL-1 was relatively highly expressed both in normal and tumor tissues (Fig. 2a, b). Taken together, this data suggested that BCL-X_L_ is the most consistently upregulated BCL-2 family member in SCCHN, although targeting BCL-X_L_ alone did not result in enhanced apoptosis in SCCHN cell lines (Fig. 1c).

### High expression levels of BCL-X_L_ and MCL-1 do not correlate with patient outcome

**Fig. 3:**
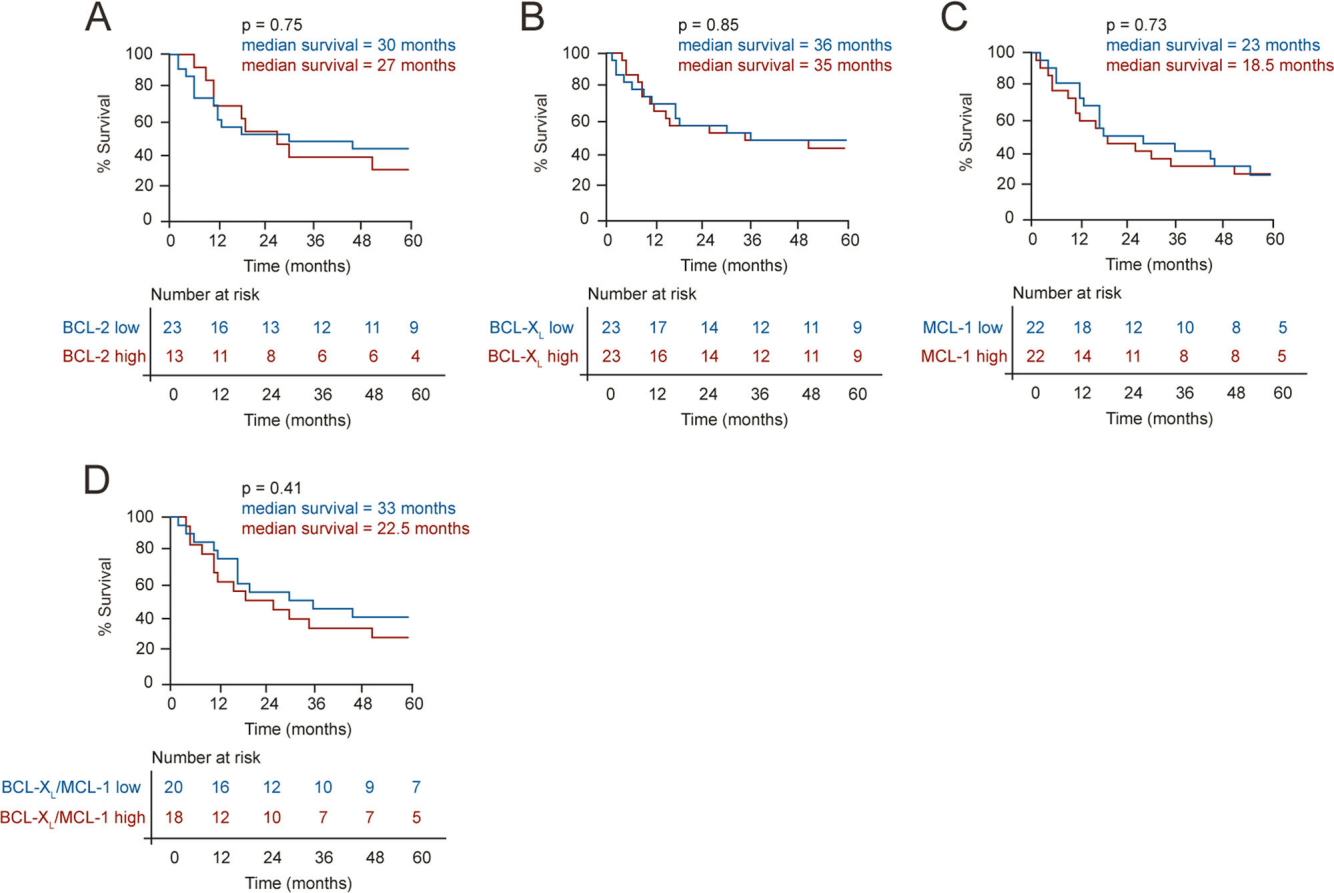
Expression levels of the antiapoptotic BCL-2 family members do not correlate with patient overall survival. Kaplan–Meier curves comparing the overall survival of patients (censored at 60 months) in the highest versus lowest quartiles for expression of **a** BCL-2, **b** BCL-X_L_, **c** MCL-1, and **d** BCL-X_L_ and MCL-1, in oral cavity tumors. Numbers at risk are displayed below each graph, to demonstrate the numbers of patients analyzed per category. Comparison of survival curves used the log-rank (Mantel–Cox) test.

No correlation was observed between expression of BCL-2, BCL-X_L_, or MCL-1 and overall survival of patients with oral cavity tumors (Fig. 3a–c). This was surprising as numerous studies have reported positive correlations between these proteins and patient outcome in different malignancies, often using antibodies that had not been validated for IHC analysis. The importance of using carefully validated antibodies was further emphasized by the stark contrast in survival plots generated using different MCL-1 antibodies (Supplementary Fig. S4). Since individual expression levels of MCL-1 and BCL-X_L_ did not correlate with patient survival, we compared the quartiles with the highest and lowest expression of both MCL-1 and BCL-X_L_ together and found no statistically significant differences in overall survival (Fig. 3d).

### Combination therapy targeting BCL-X_L_ and MCL-1 induces apoptosis in preclinical models of SCCHN

**Fig. 4:**
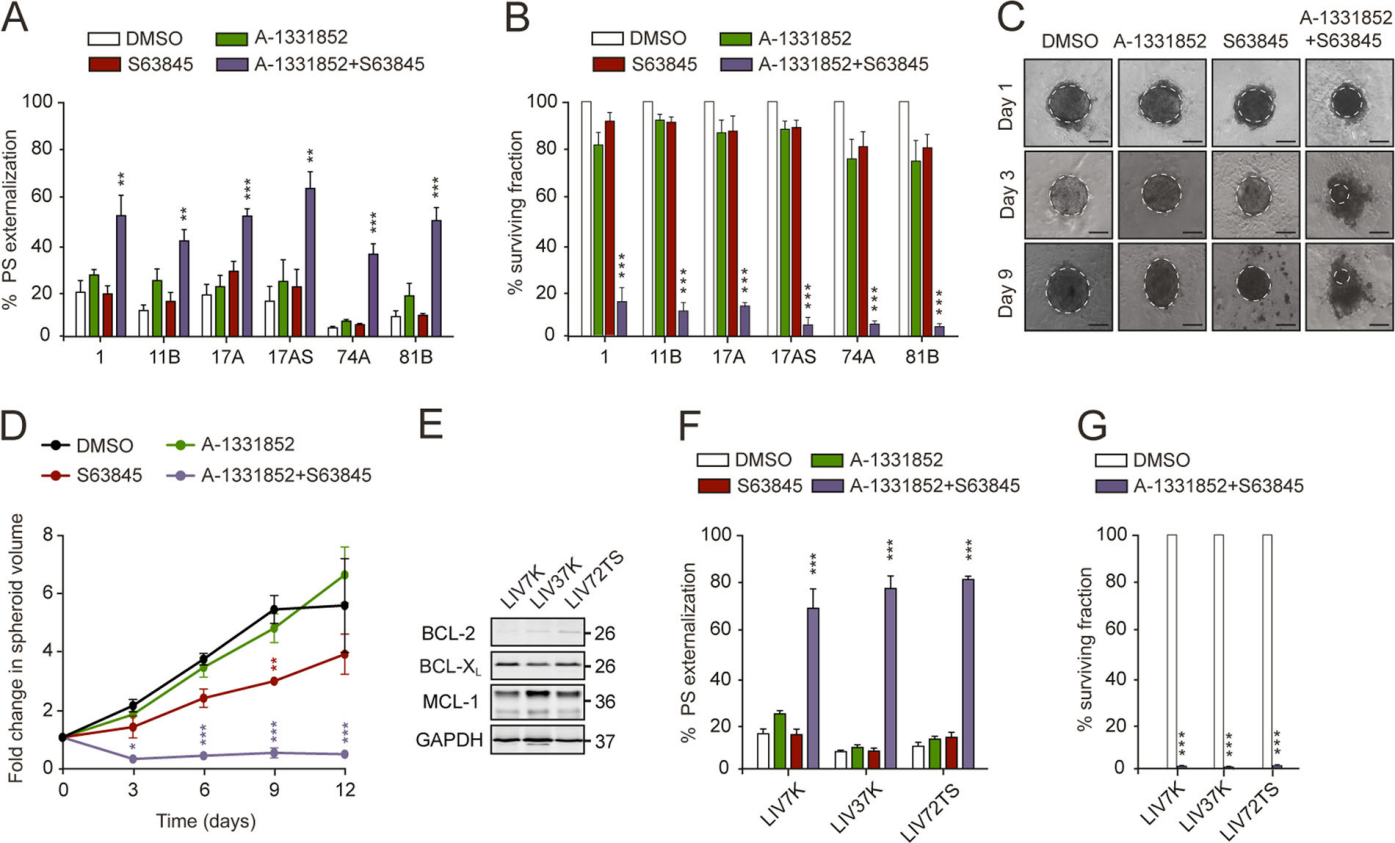
Inhibition of both MCL-1 and BCL-X_L_ induces apoptosis in SCCHN cell lines and primary cells. SCCHN cell lines exposed to a combination of A-1331852 and S63845 (100 nM each) for 24 h exhibited **a** enhanced apoptosis and **b** decreased clonogenicity, compared to the individual BH3 mimetics. **c** Phase contrast images (scale bars 100 μm), and **d** line graph to show fold change in spheroid volume over 12 days, following exposure to the specified drug(s). Dotted lines in **c** demarcate the outline of each intact spheroid. **e** Western blot analysis of antiapoptotic protein levels in SCCHN primary cells. These cells, exposed to a combination of A-1331852 and S63845 (100 nM) for 4 h exhibited **f** enhanced apoptosis and **g** decreased clonogenicity, compared to the indicated treatments. **a**, **b**, **f** One-way ANOVAs with Dunnett’s multiple comparisons tests, with a single pooled variance; **d** two-way repeated measures ANOVA with Dunnett’s multiple comparisons test; **g** unpaired, two-tailed *t* tests. Error bars = mean ± SEM of at least three independent experiments. *P < 0.05, **P < 0.01, ***P < 0.001.

Although we did not find a correlation between BCL-X_L_ and MCL-1 and patient outcome, these antiapoptotic proteins are highly expressed in the majority of SCCHN patients, suggesting their involvement in carcinogenesis. As many solid tumors depend on both BCL-X_L_ and MCL-1 for survival^30^, it raised the possibility that targeting these two proteins together may be a beneficial treatment strategy. In support of this, a combination of specific BCL-X_L_ and MCL-1 inhibitors, A-1331852 and S63845, induced marked apoptosis in all SCCHN cell lines (Fig. 4a). In contrast, exposure to the BCL-2-specific inhibitor, ABT-199, either alone or in combination with A-1331852 or S63845 did not enhance apoptosis (Supplementary Fig. S5). These results strongly suggested that these cell lines are dependent on both BCL-X_L_ and MCL-1 for survival, with little or no contribution from BCL-2. Furthermore, a combination of A-1331852 and S63845 markedly inhibited the clonogenic potential of all six SCCHN cell lines, whereas either inhibitor alone exerted little or no inhibition (Fig. 4b). Together, A-1331852 and S63845 markedly inhibited the growth of 3D tumor spheroids, which mimic the in vivo behavior of tumor cells more closely than 2D cultures^31^, whereas S63845 alone caused a modest inhibition and A-1331852 had no effect (Fig. 4c, d). Consistent with these findings, primary cell lines derived from oral cavity tumors also expressed both BCL-X_L_ and MCL-1 with little detectable BCL-2 (Fig. 4e). Furthermore, the combination of A-1331852 and S63845 induced apoptosis and inhibited the clonogenic potential of all SCCHN patient-derived cell lines (Fig. 4f, g).

### Targeting BCL-X_L_ and MCL-1 induces apoptosis in tumor tissues resected from SCCHN patients

**Fig. 5:**
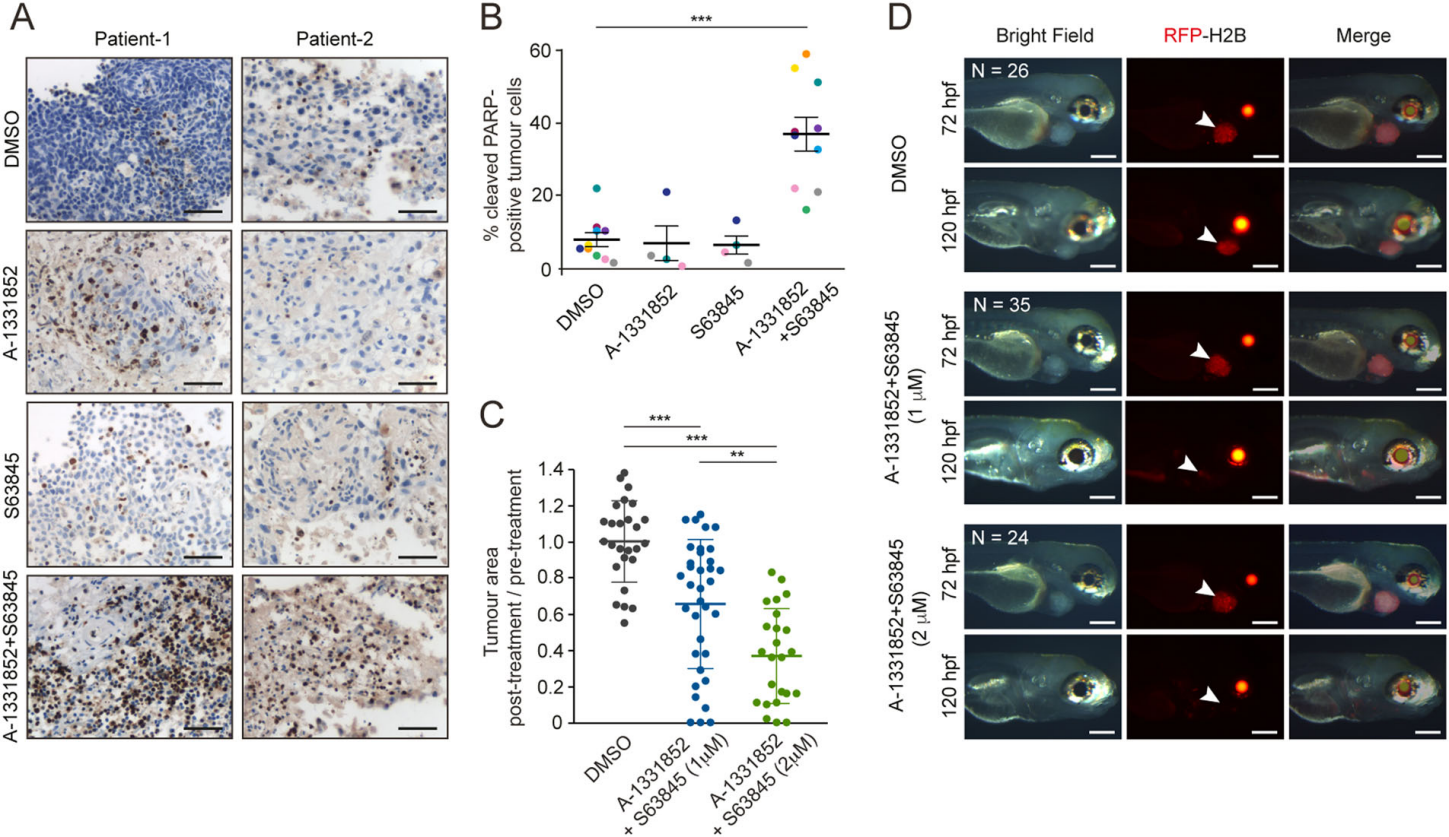
A combination of A-1331852 and S63845 induces apoptosis in SCCHN patient explants and reduces tumor burden in zebrafish xenografts. **a** Representative IHC images from two patients (scale bars 50 μm) and **b** quantitation of cleaved PARP staining in SCCHN explants from up to 10 patients treated for 48 h, as indicated. Each point in the dot plot represents one patient. Approximately, 1000–2000 cells were counted per patient, per treatment. One-way ANOVA with Dunnett’s multiple comparisons test. Error bars = mean ± SEM. ***P < 0.001. **c** Dot plots (each point represents one zebrafish) show the size of each post-treatment xenograft at 120 hpf, normalized to the mean of the DMSO-treated (control) group. One-way ANOVA with Tukey’s multiple comparisons test. Error bars = mean ± SD. **P < 0.01, ***P < 0.001. **d** Representative images (scale bars 200 μm) of zebrafish containing xenografts of UM-SCC-81B cells expressing H2B-mRFP. Arrowheads indicate the tumor cells and the effects of the treatments. Pre-treatment images are labeled 72 hpf, and images acquired following 48 h exposure to the specified drugs are labeled 120 hpf. The red fluorescence observed in the eyes is a result of a red lens reporter in the *ubiq*:secAnnexinV-mVenus fish.

To increase the translational relevance and more closely mimic the in vivo setting, tumor tissues that maintain tissue architecture as well as several aspects of the tumor microenvironment were surgically resected from SCCHN patients and exposed to A-1331852 and/or S63845, and the extent of apoptosis assessed by the appearance of cleaved PARP in apoptotic nuclei. Exposure of tumor explants to either A-1331852 or S63845 alone did not induce apoptosis, whereas the combination resulted in a significant induction of apoptosis (Fig. 5a, b).

### Targeting BCL-X_L_ and MCL-1 reduces tumor burden in zebrafish xenografts

To ascertain whether this combination was effective in vivo, a NC3Rs (National Center for Replacement, Refinement and Reduction of animals in research)-compliant zebrafish SCCHN xenograft model, which allows moderate to high throughput analyses for cancer discovery studies, was employed. BH3 mimetics have previously been administered to zebrafish embryos, with minimal toxicity^32^. Initial toxicity studies with our BH3 mimetics were performed using 3–5 day zebrafish embryos, and sublethal concentrations (1–2 μM) chosen for subsequent experiments (Supplementary Fig. S6). To establish tumors in vivo, fluorescently labeled SCCHN cells were microinjected into the pericardial cavity at 48 hpf. Following verification of the presence of fluorescent cell masses 24 h postinjection, zebrafish embryos were randomly allocated either to a DMSO only (control) group or exposed to a combination of A-1331852 and S63845 for 48 h (72–120 hpf) and final tumor area assessed (Fig. 5c, d). Dual inhibition of BCL-X_L_ and MCL-1 resulted in a significant dose-dependent reduction in tumor size compared to controls (Fig. 5c, d). These results were in agreement with the data generated using cell lines, primary cells and patient explants.

## Discussion

A major mechanism by which cancer cells evade apoptosis is overexpression of antiapoptotic BCL-2 family proteins^8^. Hematological malignancies largely depend on a single antiapoptotic member for survival, and as a result, monotherapy with BH3 mimetics has been successful^12,16,17^. However, as our study and others show, solid tumors depend on more than one BCL-2 family member for survival, and in most instances, neutralization of both BCL-X_L_ and MCL-1 is required to induce efficient apoptosis^30,33,34^.

Studies investigating the BCL-2 family of proteins in SCCHN have reached conflicting conclusions with respect to BCL-2 expression and its correlation with SCCHN patient outcome^35–46^. Whilst the basis for these discrepancies is unknown, it could be partly attributed to a lack of proper antibody validation. Using a carefully validated BCL-2 antibody (Supplementary Fig. S3), we convincingly demonstrate an overall low expression of BCL-2 (Figs. 1 and 2) and conclude that the most successful BH3 mimetic, venetoclax (ABT-199, currently used in ~146 clinical trials) is highly unlikely to be effective for the majority of SCCHN patients.

Just a handful of papers exist correlating BCL-X_L_ and MCL-1 expression with patient outcome in SCCHN^36,41,43,47,48^. Many of these studies have relied on mRNA levels rather than IHC and report either a positive or negative correlation to clinical outcome. In our study, expression of BCL-X_L_ and MCL-1 was generally higher in tumors than surrounding normal tissues, suggesting that some selectivity could be gained by targeting these proteins (Figs. 1 and 2). MCL-1 expression appeared to decrease in the oral cavity advancing front (Fig. 1), possibly due to the functional redundancy between MCL-1 and BCL-X_L_. BCL-X_L_ may play an additional role in the invasive advancing front, as it has recently been proposed to promote epithelial–mesenchymal transition and tumor metastasis^49^.

Despite high expression in tumor compared to normal tissues, expression levels of BCL-X_L_ and MCL-1 did not correlate with patient outcome in SCCHN (Figs. 1–3). This could have been confounded by heterogeneity in the adjuvant treatment (none or radiotherapy±chemotherapy) post-surgery and warrants a prospective cohort analysis. Although BCL-X_L_ and MCL-1 were not prognostic indicators in SCCHN, dual inhibition resulted in extensive apoptosis in all preclinical models including tumor explants, as well as a decreased tumor burden in zebrafish xenografts (Figs. 4 and 5). Since BCL-X_L_ and MCL-1 share a high degree of functional redundancy both in tumor survival and in normal cellular homeostasis, a combination of inhibitors targeting these proteins may result in some degree of toxicity to normal tissues. Inhibition of BCL-X_L_ results in thrombocytopenia due to platelets being dependent on BCL-X_L_ for survival^50^, whereas inhibition of MCL-1 will affect survival of neutrophils^51^, thus mandating the establishment of a therapeutic index and optimal scheduling for the drug combination, as has been achieved to manage Navitoclax-induced thrombocytopenia in patients^52^. Furthermore, it is important to note the preferential expression of both BCL-X_L_ and MCL-1 in tumor compared to the normal tissues (Figs. 1 and 2), which provides a basis for BH3 mimetics to selectively target the tumor tissue.

First-line therapy of cisplatin only modestly improves overall survival in SCCHN patients, at the cost of significant toxicity. Furthermore, many HPV^−^ SCCHN patients with locally advanced disease are simply not fit enough for cisplatin therapy, thus emphasizing the need to identify and introduce better, more targeted therapies for this malignancy. It is possible that dual inhibition of BCL-X_L_ and MCL-1 could potentially replace cisplatin in patients unable to tolerate this, or reduce the doses of radiotherapy and/or cisplatin administered to patients.

In conclusion, we convincingly demonstrate that BCL-X_L_ and MCL-1 but not BCL-2 are highly expressed in tumor tissues of SCCHN patients. The low expression or absence of BCL-2 suggests that there may be little benefit in using venetoclax to treat SCCHN. We also show that SCCHN is dependent on both BCL-X_L_ and MCL-1 for survival, which can be efficiently targeted to induce marked apoptosis and tumor shrinkage, suggesting that a combination BH3 mimetic therapy may offer significant benefits in SCCHN.

## Supplementary information

S1

S2

S3

S4

S5

S6

Supplementary Legends

## References

[1] Ferlay J (2019). Estimating the global cancer incidence and mortality in 2018: GLOBOCAN sources and methods. Int. J. Cancer.

[2] Shaw R, Beasley N (2016). Aetiology and risk factors for head and neck cancer: United Kingdom National Multidisciplinary Guidelines. J. Laryngol. Otol..

[3] The Cancer Genome Atlas Network. (2015). Comprehensive genomic characterization of head and neck squamous cell carcinomas. Nature.

[4] Alsahafi E (2019). Clinical update on head and neck cancer: molecular biology and ongoing challenges. Cell Death Dis..

[5] Lo Nigro C, Denaro N, Merlotti A, Merlano M (2017). Head and neck cancer: improving outcomes with a multidisciplinary approach. Cancer Manag. Res..

[6] Yamaoka T, Ohba M, Ohmori T (2017). Molecular-targeted therapies for epidermal growth factor receptor and its resistance mechanisms. Int. J. Mol. Sci..

[7] Rischin D (2019). Protocol-specified final analysis of the phase 3 KEYNOTE-048 trial of pembrolizumab (pembro) as first-line therapy for recurrent/metastatic head and neck squamous cell carcinoma (R/M HNSCC). J. Clin. Oncol..

[8] Youle RJ, Strasser A (2008). The BCL-2 protein family: opposing activities that mediate cell death. Nat. Rev. Mol. Cell Biol..

[9] Soderquist RS, Eastman A (2016). BCL2 inhibitors as anticancer drugs: a plethora of misleading BH3 mimetics. Mol. Cancer Ther..

[10] Oltersdorf T (2005). An inhibitor of Bcl-2 family proteins induces regression of solid tumours. Nature.

[11] Tse C (2008). ABT-263: a potent and orally bioavailable Bcl-2 family inhibitor. Cancer Res..

[12] Souers AJ (2013). ABT-199, a potent and selective BCL-2 inhibitor, achieves antitumor activity while sparing platelets. Nat. Med..

[13] Roberts AW (2016). Targeting BCL2 with Venetoclax in relapsed chronic lymphocytic leukemia. N. Engl. J. Med..

[14] DiNardo CD (2018). Safety and preliminary efficacy of venetoclax with decitabine or azacitidine in elderly patients with previously untreated acute myeloid leukaemia: a non-randomised, open-label, phase 1b study. Lancet Oncol..

[15] Leverson JD (2015). Exploiting selective BCL-2 family inhibitors to dissect cell survival dependencies and define improved strategies for cancer therapy. Sci. Transl. Med..

[16] Lucas CM (2016). High CIP2A levels correlate with an antiapoptotic phenotype that can be overcome by targeting BCL-XL in chronic myeloid leukemia. Leukemia.

[17] Kotschy A (2016). The MCL1 inhibitor S63845 is tolerable and effective in diverse cancer models. Nature.

[18] Caenepeel S (2018). AMG 176, a selective MCL1 inhibitor, is effective in hematologic cancer models alone and in combination with established therapies. Cancer Discov..

[19] Tron AE (2018). Discovery of Mcl-1-specific inhibitor AZD5991 and preclinical activity in multiple myeloma and acute myeloid leukemia. Nat. Commun..

[20] dos Santos LV, Carvalho AL (2011). Bcl-2 targeted-therapy for the treatment of head and neck squamous cell carcinoma. Recent Pat. Anticancer Drug Discov..

[21] Li R (2009). ABT-737 synergizes with chemotherapy to kill head and neck squamous cell carcinoma cells via a Noxa-mediated pathway. Mol. Pharmacol..

[22] Gilormini M (2016). Preferential targeting of cancer stem cells in the radiosensitizing effect of ABT-737 on HNSCC. Oncotarget.

[23] Bankhead P (2017). QuPath: open source software for digital pathology image analysis. Sci. Rep..

[24] Morsch M (2015). In vivo characterization of microglial engulfment of dying neurons in the zebrafish spinal cord. Front. Cell Neurosci..

[25] Chapman A (2014). Heterogeneous tumor subpopulations cooperate to drive invasion. Cell Rep..

[26] Butterworth M, Pettitt A, Varadarajan S, Cohen GM (2016). BH3 profiling and a toolkit of BH3-mimetic drugs predict anti-apoptotic dependence of cancer cells. Br. J. Cancer.

[27] Roncador G (2016). The European antibody network’s practical guide to finding and validating suitable antibodies for research. MAbs.

[28] Goodman SL (2018). The path to VICTORy—a beginner’s guide to success using commercial research antibodies. J. Cell Sci..

[29] Perciavalle RM (2012). Anti-apoptotic MCL-1 localizes to the mitochondrial matrix and couples mitochondrial fusion to respiration. Nat. Cell Biol..

[30] Greaves G (2019). BH3-only proteins are dispensable for apoptosis induced by pharmacological inhibition of both MCL-1 and BCL-XL. Cell Death Differ..

[31] Melissaridou S (2019). The effect of 2D and 3D cell cultures on treatment response, EMT profile and stem cell features in head and neck cancer. Cancer Cell Int..

[32] Li Z, He S, Look AT (2019). The MCL1-specific inhibitor S63845 acts synergistically with venetoclax/ABT-199 to induce apoptosis in T-cell acute lymphoblastic leukemia cells. Leukemia.

[33] Cho SY (2017). A novel combination treatment targeting BCL-XL and MCL1 for KRAS/BRAF-mutated and BCL2L1-amplified colorectal cancers. Mol. Cancer Ther..

[34] Weeden CE (2018). Dual inhibition of BCL-XL and MCL-1 is required to induce tumour regression in lung squamous cell carcinomas sensitive to FGFR inhibition. Oncogene.

[35] Wilson GD (1996). Bcl-2 expression correlates with favourable outcome in head and neck cancer treated by accelerated radiotherapy. Anticancer Res..

[36] Pena JC, Thompson CB, Recant W, Vokes EE, Rudin CM (1999). Bcl-xL and Bcl-2 expression in squamous cell carcinoma of the head and neck. Cancer.

[37] Yuen AP (2002). Clinicopathologic significance of bcl-2 expression in the surgical treatment of oral tongue carcinoma. Eur. J. Surg. Oncol..

[38] Wilson GD (2001). Bcl-2 expression in head and neck cancer: an enigmatic prognostic marker. Int. J. Radiat. Oncol. Biol. Phys..

[39] Trask DK (2002). Expression of Bcl-2 family proteins in advanced laryngeal squamous cell carcinoma: correlation with response to chemotherapy and organ preservation. Laryngoscope.

[40] Hotz MA (1999). Spontaneous apoptosis and the expression of p53 and Bcl-2 family proteins in locally advanced head and neck cancer. Arch. Otolaryngol. Head Neck Surg..

[41] Mallick S (2009). Human oral cancers have altered expression of Bcl-2 family members and increased expression of the anti-apoptotic splice variant of Mcl-1. J. Pathol..

[42] Pallavi N, Nalabolu GRK, Hiremath SKS (2018). Bcl-2 and c-Myc expression in oral dysplasia and oral squamous cell carcinoma: an immunohistochemical study to assess tumor progression. J. Oral Maxillofac. Pathol..

[43] Ow TJ (2019). Optimal targeting of BCL-family proteins in head and neck squamous cell carcinoma requires inhibition of both BCL-xL and MCL-1. Oncotarget.

[44] Friedman M (1997). Prognostic significance of Bcl-2 expression in localized squamous cell carcinoma of the head and neck. Ann. Otol. Rhinol. Laryngol..

[45] Lo Muzio L (2005). Bcl-2 as prognostic factor in head and neck squamous cell carcinoma. Oncol. Res..

[46] Redondo M (2006). Expression of the antiapoptotic proteins clusterin and bcl-2 in laryngeal squamous cell carcinomas. Tumour Biol..

[47] Zhang K (2014). Bcl-xL overexpression and its association with the progress of tongue carcinoma. Int. J. Clin. Exp. Pathol..

[48] Palve V, Mallick S, Ghaisas G, Kannan S, Teni T (2014). Overexpression of Mcl-1L splice variant is associated with poor prognosis and chemoresistance in oral cancers. PLoS ONE.

[49] Choi S (2016). Bcl-xL promotes metastasis independent of its anti-apoptotic activity. Nat. Commun..

[50] Mason KD (2007). Programmed anuclear cell death delimits platelet life span. Cell.

[51] Murphy MP, Caraher E (2015). Mcl-1 is vital for neutrophil survival. Immunol. Res..

[52] Gandhi L (2011). Phase I study of Navitoclax (ABT-263), a novel Bcl-2 family inhibitor, in patients with small-cell lung cancer and other solid tumors. J. Clin. Oncol..

