# Supplementary figures and images for: Exploring the potential of BH3 mimetic therapy in squamous cell carcinoma of the head and neck
Source: Cell Death Dis. 2019 Dec 4;10(12):912. doi: 10.1038/s41419-019-2150-8 (PMC6892862; doi:10.1038/s41419-019-2150-8)

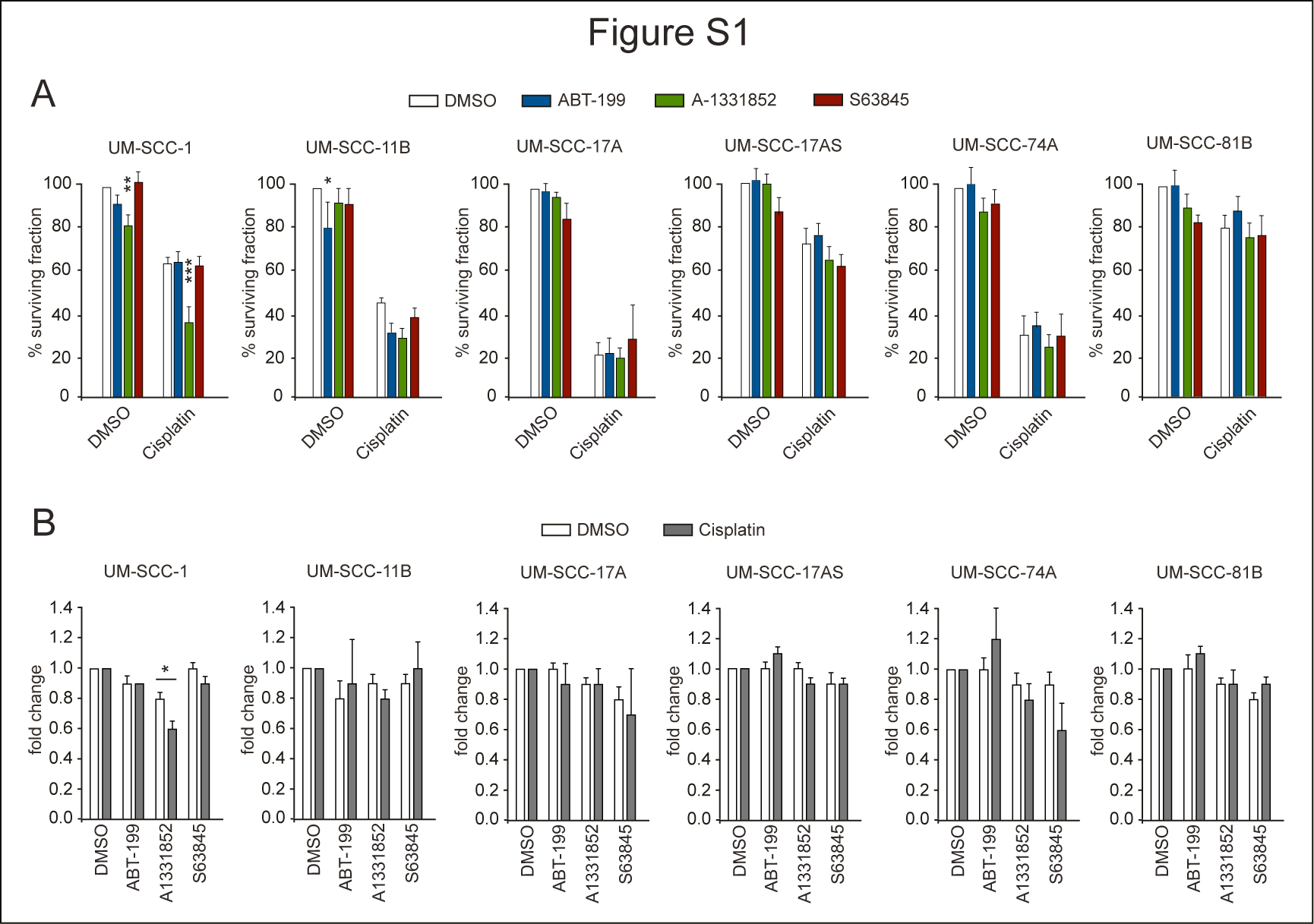

Supplement: Supplementary file 1 — S1 [file 41419_2019_2150_MOESM1_ESM.tif]

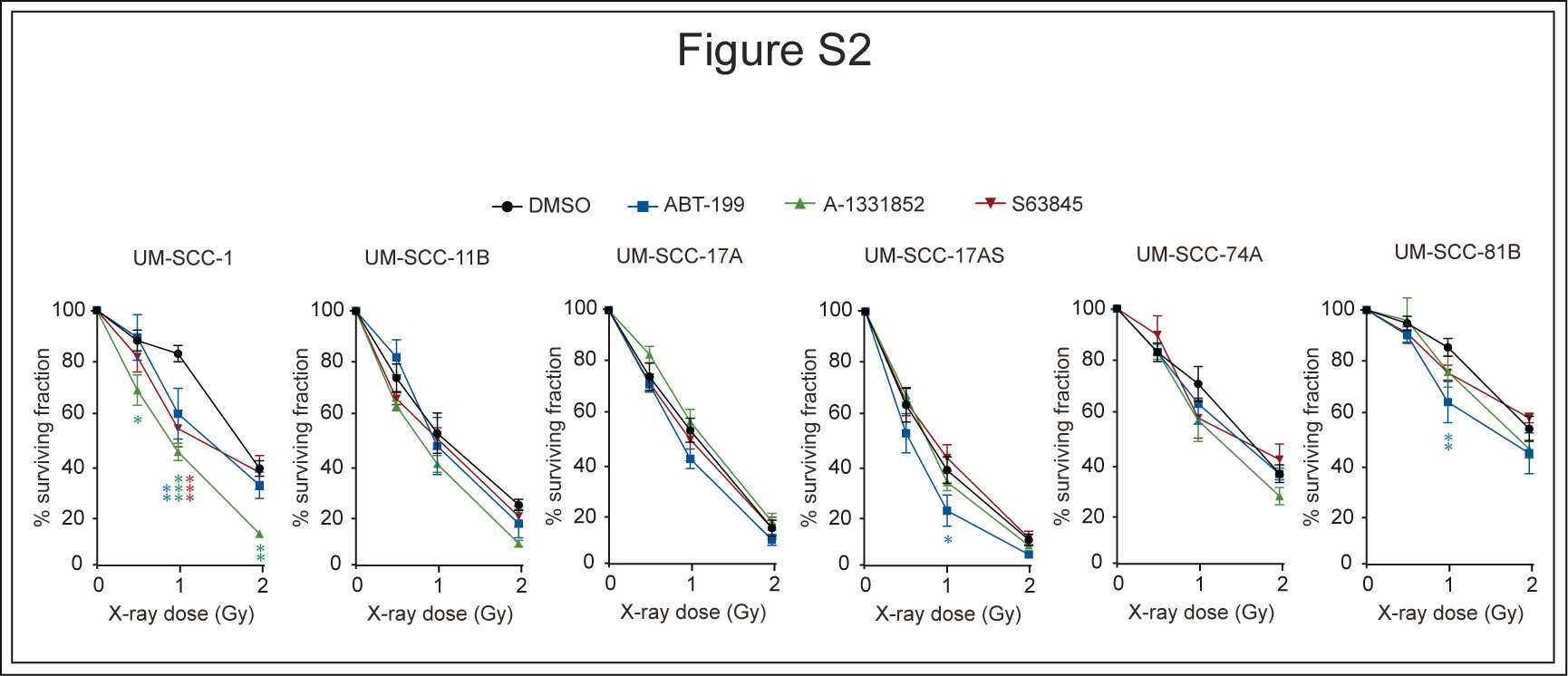

Supplement: Supplementary file 2 — S2 [file 41419_2019_2150_MOESM2_ESM.tif]

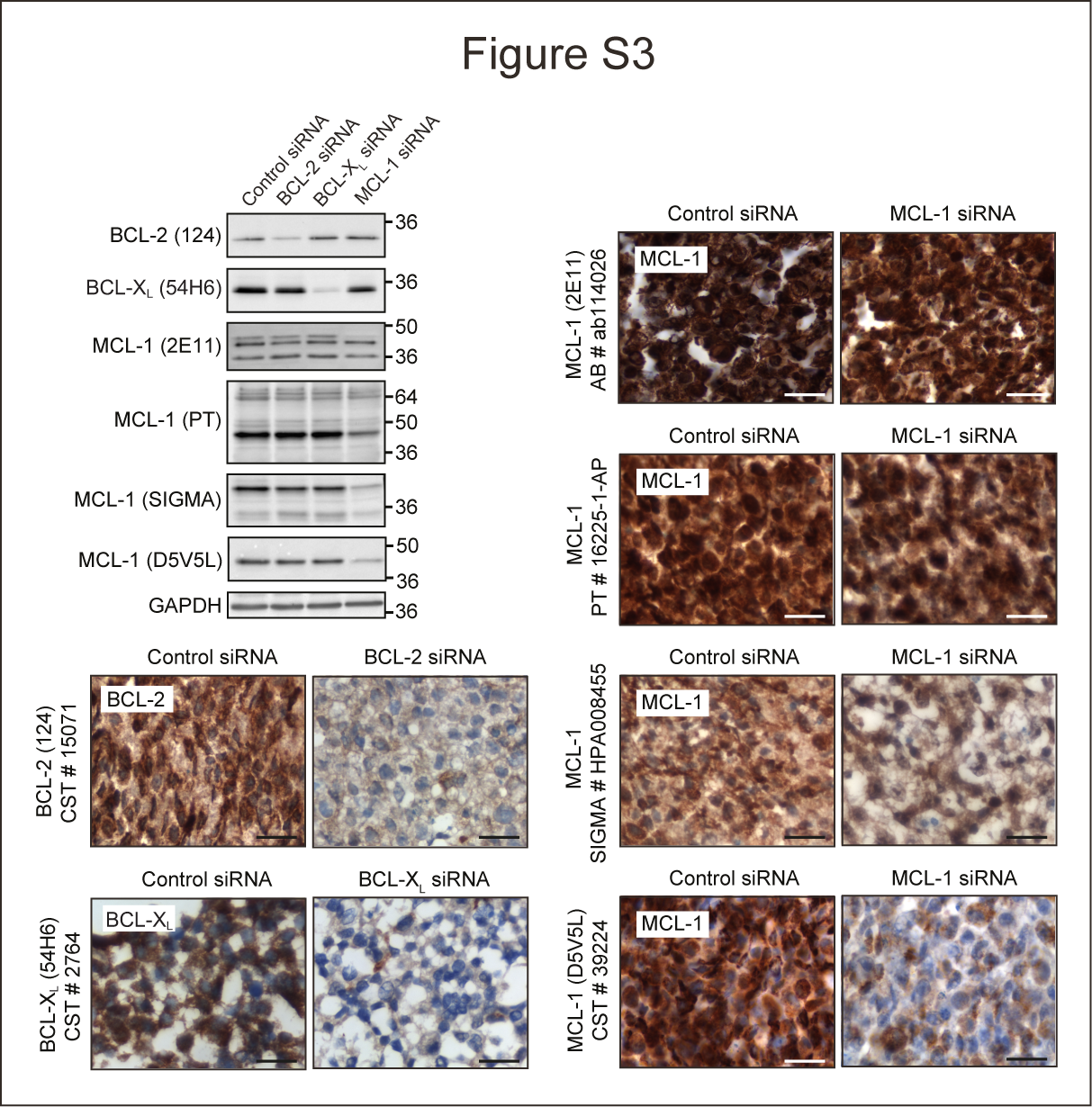

Supplement: Supplementary file 3 — S3 [file 41419_2019_2150_MOESM3_ESM.tif]

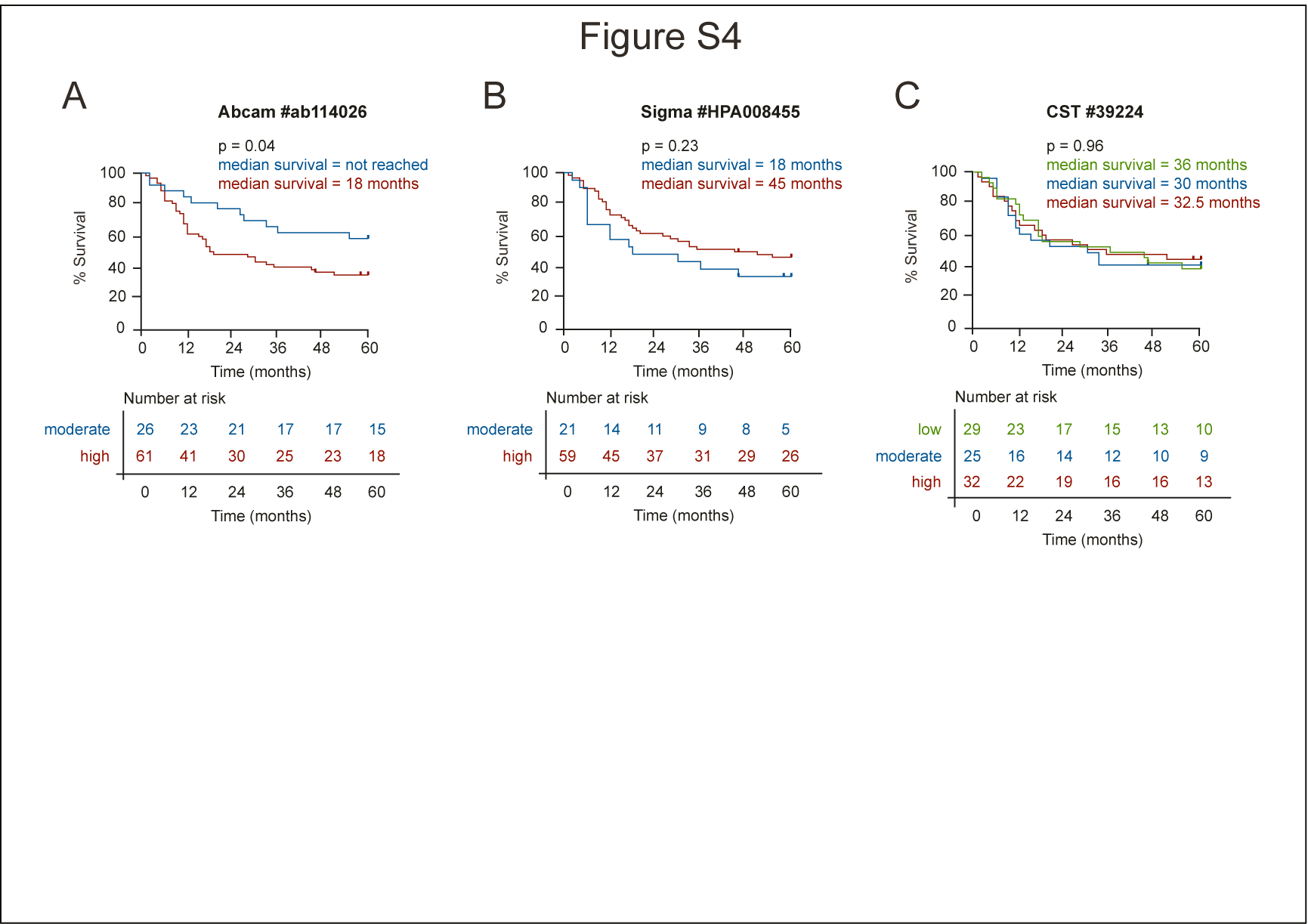

Supplement: Supplementary file 4 — S4 [file 41419_2019_2150_MOESM4_ESM.tif]

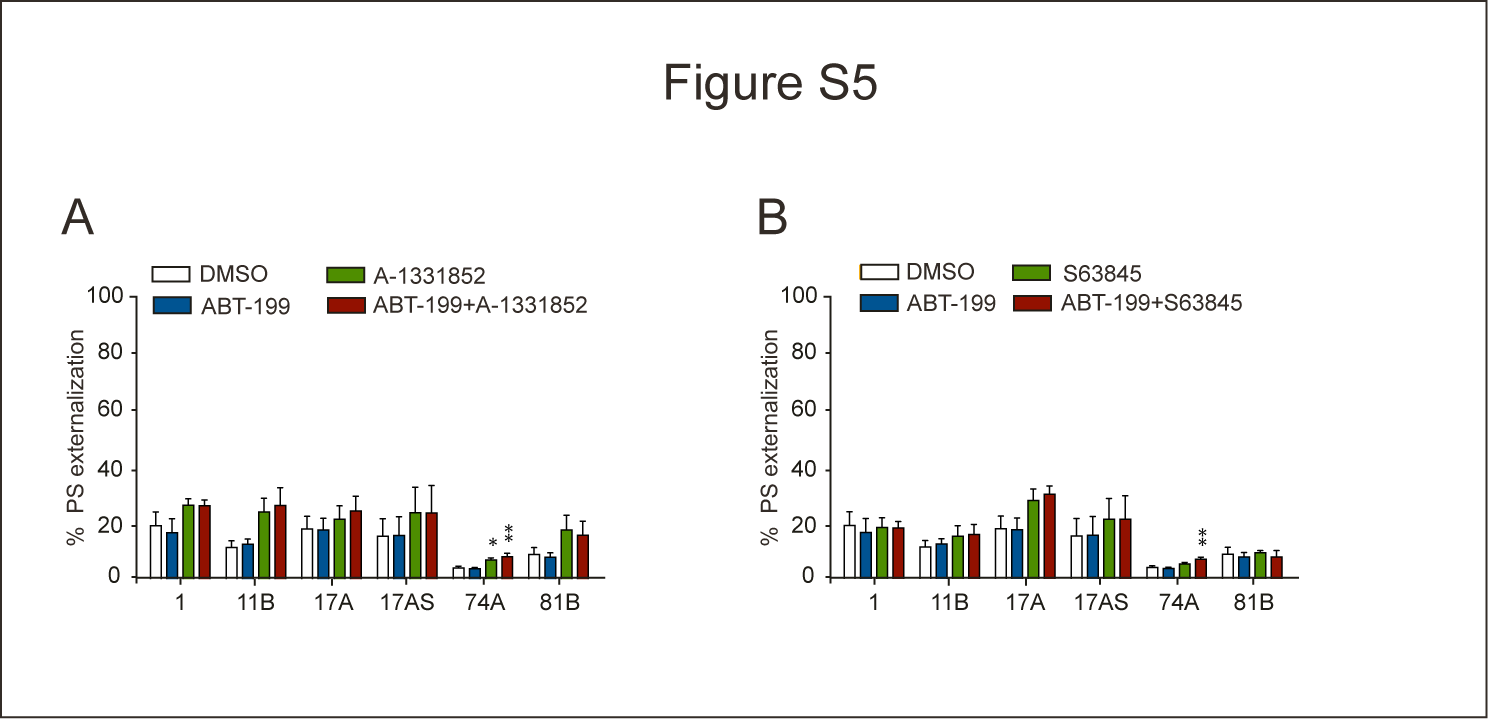

Supplement: Supplementary file 5 — S5 [file 41419_2019_2150_MOESM5_ESM.tif]

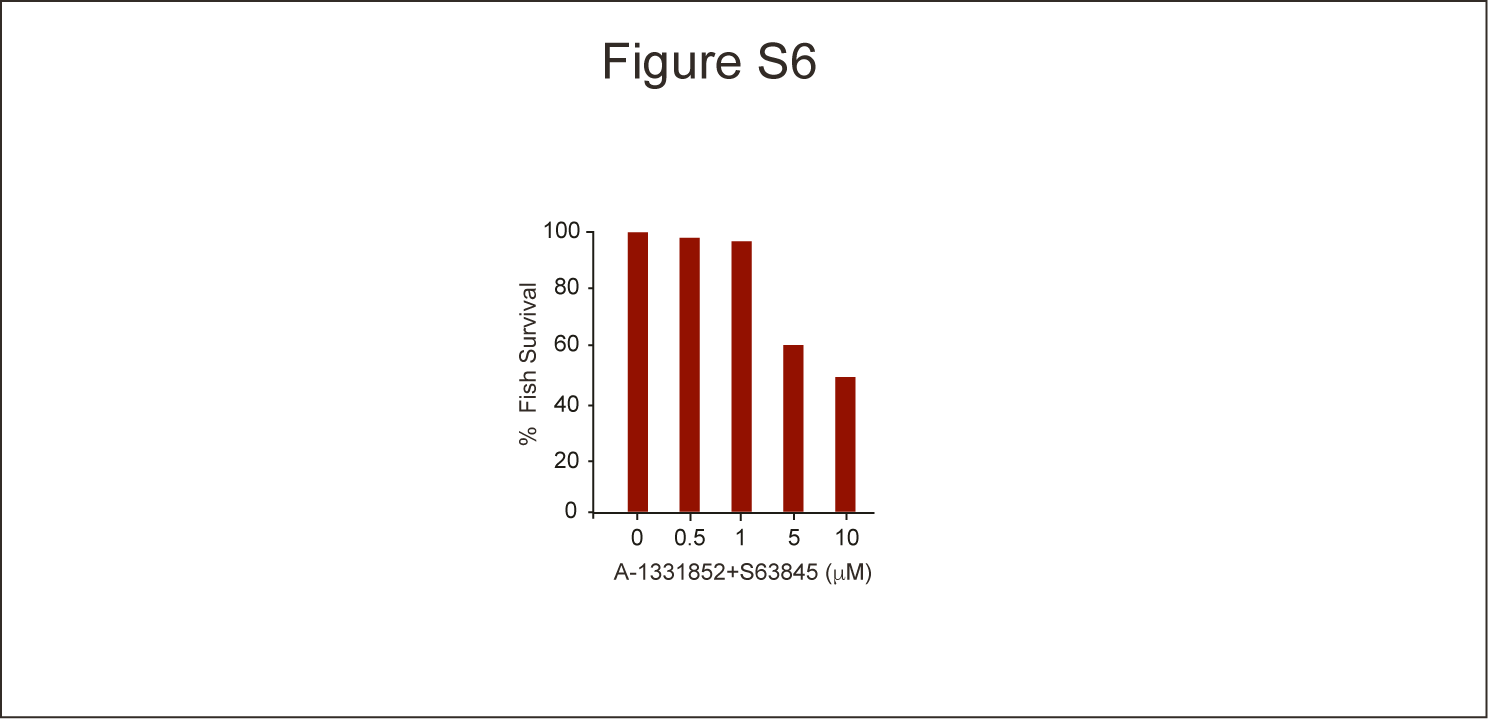

Supplement: Supplementary file 6 — S6 [file 41419_2019_2150_MOESM6_ESM.tif]
