# Supplementary material for: Exploring the potential of BH3 mimetic therapy in squamous cell carcinoma of the head and neck
Source: Cell Death Dis. 2019 Dec 4;10(12):912. doi: 10.1038/s41419-019-2150-8 (PMC6892862; doi:10.1038/s41419-019-2150-8)
Supplement: Supplementary file 7 — Supplementary Legends [file 41419_2019_2150_MOESM7_ESM.docx]

**Supplementary Figure Legends**

**Supplementary Figure S1. Targeting the individual members of the BCL-2 family does not appear to synergize with cisplatin in most of the SCCHN cell lines.** **A**, Clonogenic survival of SCCHN cell lines exposed to the indicated BH3 mimetics (100 nM), alone or in combination with cisplatin (1 μM), normalized to the DMSO-treated control. Two-way ANOVA with Dunnett’s multiple comparisons test. **B**, Fold change between each BH3 mimetic and the DMSO control (white bars) was compared with the fold change between each BH3 mimetic plus cisplatin and cisplatin only (grey bars). Multiple unpaired t tests, with false discovery rate determined using the two-stage linear step-up approach of Benjamini, Krieger and Yekutieli with Q=1 %, *P<0.05, **P<0.01, ***P<0.001. Error bars=Mean±SEM.

**Supplementary Figure S2.** **Targeting the individual members of the BCL-2 family does not appear to synergize with irradiation in most of the SCCHN cell lines.** Clonogenic survival of SCCHN cell lines following exposure to the indicated BH3 mimetics (100 nM), alone (0 Gy) or in combination with 0.5, 1 or 2 Gy x-ray irradiation. Two-way ANOVAs with Dunnett’s multiple comparisons tests, *P<0.05, **P<0.01, ***P<0.001. Error bars=Mean±SEM.

**Supplementary Figure S3. Validation of the different antibodies for use in IHC analysis.** Western blots of UM-SCC-74A cells used to generate pellets for IHC. Images show IHC staining of sections of paraffin-embedded UM-SCC-74A cell pellets, which had been transfected with the indicated siRNA (10 nM) for 72 h. Antibodies – including clones (in brackets) and product codes (#) – are detailed adjacent to images. CST, Cell Signaling Technology; AB, Abcam; PT, Proteintech; SIGMA, Sigma-Aldrich. Scale bars 25 μm.

**Supplementary Figure S4. Survival plots vary significantly based on the different MCL-1 antibodies used in IHC analysis.** Kaplan-Meier graphs showing overall survival of patients, up to 5 years, stratified according to the expression levels of MCL-1 in oral cavity tumor cores, determined using three different MCL-1 antibodies: **A**, Abcam ab114026, **B**, Sigma HPA008455, and **C**, CST 39224. Numbers at risk are displayed below each graph, to demonstrate the numbers of patients analyzed per category. Log-rank (Mantel-Cox) test was used to compare survival curves.

**Supplementary Figure S5. ABT-199, alone or in combination with A-1331852 or S63845, does not result in apoptosis of the SCCHN cell lines.** Apoptosis induction, assessed by flow cytometry of PS externalization, in SCCHN cell lines exposed to **A,** ABT-199 (100 nM), alone or in combination with A-1331852 (100 nM) and **B**, ABT-199 (100 nM), alone or in combination with S63845 (100 nM). One-way ANOVAs with Dunnett’s multiple comparisons tests, with a single pooled variance, *P<0.05, **P<0.01. Error bars=Mean±SEM.

**Supplementary Figure S6. Toxicity plot in zebrafish to determine the appropriate concentration of BH3 mimetics for dosing.** Graph depicting the overall survival in fish (out of approximately 100 fish per dose) at 120 hpf, following exposure to increasing doses of the combination of A-1331852 and S63845 at 72 hpf.
